# A descriptive study of the participation of children and adolescents in activities outside school

**DOI:** 10.1186/s12887-016-0623-9

**Published:** 2016-07-08

**Authors:** Christine Imms, Elspeth Froude, Brooke Adair, Nora Shields

**Affiliations:** Centre for Disability and Development Research, Australian Catholic University, Level 2, Daniel Mannix Building, 17 Young Street, Fitzroy, 3065 VIC Australia; Murdoch Childrens Research Institute, Melbourne, Australia; CanChild Centre for Childhood Disability Research, Hamilton, Canada; School of Allied Health, Australian Catholic University, Sydney, Australia; School of Allied Health, La Trobe University, Melbourne, Australia; Northern Health, Melbourne, Australia

**Keywords:** Participation, Leisure, Typical development, Children, Adolescents, Normative data

## Abstract

**Background:**

Knowledge about patterns of participation can be used to highlight groups of children and adolescents with low attendance, or low involvement in activities and who may therefore be at risk of mental or physical health concerns. This study used the *Children’s Assessment of Participation and Enjoyment* (CAPE) and the *Preferences for Activity of Children* (PAC) to describe the patterns of participation of children and adolescents in activities outside mandated school in Victoria, Australia.

**Methods:**

A cross-sectional survey of Victorian children and adolescents was conducted. Eligible participants were aged 6 to 18 years, enrolled in mainstream schools, with sufficient English language skills to complete the questionnaires. Parents of participants completed a demographic questionnaire. Sample representativeness was assessed against Victorian population statistics for gender, school type, language spoken at home and socio-economic status. Data for the CAPE and PAC were summarised using descriptive statistics. Patterns of activity diversity by age were assessed using curve estimation, with additional analyses to describe differences between genders.

**Results:**

Of 9337 potential participants targeted through school advertising, 512 agreed (5.5 % consent rate), and 422 questionnaires were returned (82.4 % response rate). The sample was representative in terms of gender and language. Compared to the Victorian population, a slightly higher proportion of participants attended Government and Catholic schools and there was evidence of marginally greater socioeconomic resources than the population average.

A broad range of recreational, active physical, social, skill-based and self-improvement activities were completed by all age groups. There was a reduction in the number and enjoyment of recreational activities with increasing age. In contrast, there was relative stability in intensity, frequency and preference scores across the age-groups for all activity types. Female participants typically took part in more activities (higher diversity scores), more intensely, with higher enjoyment and had higher preferences for each activity type than males, with the exception of active physical activities.

**Conclusions:**

This study provides evidence of the participation patterns of typically developing children and adolescents in activities outside school. The findings have implications for researchers, clinicians and educators for comparative purposes and to inform future research.

**Electronic supplementary material:**

The online version of this article (doi:10.1186/s12887-016-0623-9) contains supplementary material, which is available to authorized users.

## Background

Successful participation of children and adolescents in home, school and community settings is argued to be the ultimate goal of both health and education services [1]. Defined as “involvement in a life situation” [2] (page 9) participation is known to be a complex multidimensional concept that has both an attendance and involvement component [3]. The attendance component relates to children ‘being there’, that is, attending a range of activities at a desired frequency. The involvement component relates to the ‘in-the-moment’ experience of participation and might include affect, motivation or social connection [3]. Although not explicitly defined, life situations have been described as frequently occurring routines that are complex, situated in social contexts and directed towards meaningful goals [4]. The focus of the current study was on children and adolescent’s participation patterns in activities outside mandated school: this ‘life situation’ can include a broad range of home, work, or community activities.

The mandated school day in Australia is approximately 6 h (5 days/week), with outside-school time on school days approximating 4–5 h, and 9–10 h on non-school days (excluding sleep and personal care) [5]. As outside school leisure, or ‘free’ time, constitutes a considerable portion of the day, activity choice has important implications for the physical and mental wellbeing of children and adolescents. A rising prevalence of mental health concerns [6], obesity and Type 1 diabetes in Australian children is a national concern [7], leading to prioritisation of these public health issues [8]. The majority (>80 %) of Australian children do not engage in sufficient daily physical activity to meet the guidelines, and, in addition, more than 70 % exceed recommended daily sedentary screen-time activity [9]. Physical activity participation is of particular interest because it can confer immediate fitness benefits as well as long term mental and physical wellbeing [9].

Given outside school activities are diverse, participation patterns are of interest. Patterns of participation can be considered from the perspective of the constellation of types of activities undertaken (e.g., social or physically active) and the distribution of participation in these activity types in terms of attendance (e.g., range of activities or frequency of attending) and involvement (e.g., enjoyment of the activity). Knowledge about patterns of participation can be used to highlight groups of children and adolescents with concerning distributions of activity attendance, or low levels of involvement. There is current evidence describing the participation of children in physical or cultural activities [9, 10] and those considered detrimental to health (e.g., smoking) [6]. However, data typically reports on proportions of children taking part in particular activities, and/or time spent, with little data available to describe the broader aspects of participation, such as the overall range of activities children undertake, the fit between preferences and activities undertaken, or their enjoyment and involvement in activities.

There is a growing imperative for health services to determine if interventions provided to children with chronic health conditions, such as obesity or diabetes, and long term disability improve participation in life situations in addition to changing impairments at the level of body structure or function, and activity performance [2]. Since publication of the International Classification of Functioning Disability and Health (ICF) [11] in 2001, and the Children and Youth version (ICF-CY) [2], considerable attention has been given to understanding participation outcomes for children with a disability. Clear evidence is available describing participation restrictions in those with physical disabilities such as cerebral palsy [12–14]. A broader population approach is required however, to understand children and adolescents’ patterns of participation from which to target and quantify changes in participation arising from health and educational interventions. Use of valid and reliable outcome measures to build knowledge of the expected patterns of participation of children and adolescents without impairments can also provide evidence of the age and gender effects on participation in activities outside school.

The *Children’s Assessment of Participation and Enjoyment* (CAPE) and its companion questionnaire the *Preferences for Activity of Children* (PAC), provide valid and reliable measures of participation in activities outside mandated school [15] for children and adolescents with and without impairments. The utility of the CAPE and PAC has been substantiated through their successful use in research and practice worldwide [13, 16–21]. Several studies have compared the participation of children with disability to matched and unmatched samples of typically developing children [22–24]. Although these studies provide important information about potential determinants of participation, the limited data provided for the children and adolescents with typical development limits the utility of the data. Evaluation of the participation patterns in activities outside school of a representative sample of children and adolescents without impairments will enhance our understanding of young people’s participation and potentially assist in identifying areas for intervention for those at risk of reduced or unhealthy participation.

The primary aim of this study was to generate descriptive data for the CAPE and PAC to provide evidence of the patterns of participation of a representative sample of children and adolescents aged 6 to 18 years, without impairment, in activities outside mandated school in Victoria, Australia. Our secondary aim was to describe the patterns of participation by age and gender.

## Methods

### Design

A cross-sectional survey of children and adolescents from Victoria, Australia was conducted. Ethical approval was obtained from the Human Research Ethics Committees of La Trobe University (FHEC 09-43), Australian Catholic University (2012 125 V), Department of Education and Early Childhood Development (RIS09083) and the Catholic Education Office (GE10/0009 1635). Information letters were prepared for both parents and children to explain the study and a combined consent form that required the child and the parent’s written agreement was received prior to researchers providing the questionnaires.

### Participants

Eligible participants were children and adolescents aged 6 to 18 years, who were enrolled in mainstream schools in Victoria, Australia, with sufficient English language skills (as identified by their parents) to complete the study questionnaires. Because we wished to understand the participation patterns of those without long-term impairments, children and adolescents, who attended a special school and those with a physical, cognitive or developmental disability, as defined by their need for an integration aid to participate in mainstream school, were excluded.

### Outcome measures

Participation was measured using the CAPE and PAC questionnaires [15]. The CAPE is a 55 item questionnaire in which participants report the activities they have engaged in outside school within the previous 4 months (diversity). The questionnaire also asks for details on relative intensity (intensity), where particular activities took place (location), who the activity was performed with (companionship) and the child’s level of enjoyment (enjoyment) [15]. Participation intensity is measured by considering how often a child performs all possible activities. Participation frequency was also assessed in this study by measuring the frequency of participation in the activities actually undertaken [25]. The PAC asks about activity preferences (preference) for the same 55 items regardless of whether the respondents participated in them. The 55 activities included in the CAPE and PAC can be grouped into five activity types: recreational (e.g., playing board or card games; playing with pets), active physical (e.g., doing martial arts; gardening; playing sport), social (e.g., going to a live event; going to a party), skill-based (e.g., doing gymnastics; taking art lessons) and self-improvement (e.g., doing a religious activity; going to a public library) activities.

Good evidence is available for the validity of the CAPE and PAC for children both with and without disability [15, 25, 26]. Adequate test-retest reliability has been reported for children aged 6 to 14 years for the informal and formal activity type sub-scales (ICCs: 0.64-0.86) and the intensity and diversity scores for the activity sub-scales (ICCs: 0.67-0.81). Lower levels of reliability were reported for the enjoyment scores of the activity sub-scales (ICCs: 0.12-0.81) [15]. The surveys were completed by participant self-report, with or without assistance from parent/guardian, teacher or other person.

Given the wide range of ages in the sample, and to support consistency of data collection, parents of participants were asked to complete a demographic questionnaire (see Additional file 1). This questionnaire included questions about the participant’s gender, date of birth, address, school, year level, country of birth, parents’ country of birth, languages spoken at home, need for an integration aid and if there had been any significant events in the past 4 months that may have influenced the usual participation of the child. Socio Economic Index for Areas (SEIFA) advantage/disadvantage scores (at collector district level) were extracted from the Australian Bureau of Statistics (ABS) census database [27]. Higher SEIFA scores indicate greater socioeconomic and resource advantage.

### Procedure

The participants were recruited using stratified random and convenience sampling with the aim of achieving a representative sample of children and adolescents without long-term disability. Random methods were implemented first, and supplemented with convenience methods to increase the pool of participants, particularly those aged 15 to 18 years. Children and adolescents were invited to participate if their school was selected and agreed to distribute information about the study. School-based sampling was chosen to ensure a broad reach of the study and with the goal of including both metropolitan and rural schools. Rural schools represent approximately 46 % of all schools in Victoria, providing services to 29 % of the state’s students [28]. Victorian schools were identified using a combination of stratified random sampling (*n* = 20 Government schools) or methods of convenience (*n* = 1 Government; 2 Catholic; 1 Independent). Random sampling was performed using a complete database of Victorian Government schools and implemented according to the Like Schools Group ranking of each Government school, where the schools are ranked from 1–9 based on the amount of government assistance they receive. Twenty Government schools were randomly selected using a random numbers table from three strata (a high, middle or low Like Schools Group rank). Schools that agreed to participate either consented to researchers or teachers conducting an in-school data collection session for groups of participants (*n* = 3 schools) or to participants receiving individual mail-out survey packages. Those who returned signed consents were provided with the survey package.

The CAPE and PAC describe participation during the 4 month period preceding completion of the questionnaires, therefore seasonal variation is possible. For consistency, we attempted to collect data during the last 6 months of the year. While the majority of the sample completed assessments during this time, approximately 40 % were performed during the first half of the year.

### Study sample

We aimed to recruit a representative sample of children and adolescents of sufficient size to address our aims. No formal sample size calculations were performed. Instead, we considered aspects of the central-limit theorem to aid statistical analyses, and aimed for at least 30 participants per age group to increase the likelihood that the data would be normally distributed [29, 30].

### Data analysis

Demographic characteristics were summarised using descriptive statistics. To assess if the sample was representative of the Victorian population in terms of gender, school type and language background, comparisons were made with data provided by the Department of Education and Early Childhood Development [28, 31] using chi-squared statistics. The SEIFA scores were not normally distributed and were therefore compared with the Australian mean (1000) and standard deviation (SD) (100) [32] using a One-Sample Wilcoxon Signed Rank Test.

Activity type (recreation, active physical, social, skill-based and self-improvement) scores for the participation dimensions of diversity, intensity, enjoyment and preference were calculated according to instructions from the CAPE/PAC manual, including instructions regarding missing data. Scores for each activity type were calculated when there was ≥ 80 % of the data available for that sub-scale [15]. Some children did not participate in any of the activities from a particular activity type; in these instances the intensity, frequency and enjoyment scores for that activity type were not calculated. Frequency scores were calculated according to Imms [25]. Data for these five dimensions were summarised using means and standard deviations (SDs). Higher scores for each domain indicated participation in more diverse sets of activities and greater intensity, frequency, levels of enjoyment and preference for that activity. Results are presented by age group (years) and gender. Visual inspection of the distribution of the data and the similarity between the median and mean scores for many of the sub-categories suggested normality. Therefore, consistent with the recommendations of the developers of the tool [23, 33], data were summarised using means and SDs.

Curve estimations were applied to diversity scores to understand the patterns of participation across activity types according to age and gender. Based on visual observation, linear and quadratic approaches were selected as potential options. The most appropriate model was chosen based on the significance level of the model. Where both models fitted the data, the simplest model was chosen as the best fit, to aid in interpretation of the patterns.

Scores obtained from the location and companionship domains were collapsed to create dichotomous variables: “performed activities at home/performed activities not at home”; and “performed activities alone/performed activities with someone else” [24]. These domains were then summarised using proportions and the overall mean and SD for each proportion reported for the grouped data. Alpha was set at 0.05 for all statistical analyses, and all data were analysed using IBM SPSS Statistics Version 22.0.

## Results

Of 9337 children and adolescents invited to participate, 512 agreed (5.5 % consent rate), and of these 422 questionnaires were returned (82.4 % response rate). Our sample size targets were met for each age group except the 15 to 18 year olds (Table 1). Due to missing data we were unable to calculate a score for the intensity and/or frequency and/or enjoyment of one or more of the activity types for 14 participants (all aged 11 to 18 years). The study sample had similar characteristics to the Victorian population with regard to gender distribution and proportion for whom a language other than English was spoken at home [28, 31]. Compared to the Victorian population, there was an over-representation of participants attending Government and Catholic schools; the proportion in Independent schools was lower, as was those attending rural schools. Eleven schools (46 %), contributed *n* = 86 (20.5 %) participants from rural areas, including southern and central/inland Victoria. The number of students participating from each school ranged from 1–108 (mean = 17.5; SD = 22.0). The SEIFA scores were half a SD higher than the population mean suggesting participants may have had marginally greater socioeconomic resources than the population average (See Table 1).

**Table 1:** Participant demographics

	Proportions in study sample compared to population data		
	Study sample (*n* = (%))	Victorian population (%)	*p*-value ^a^
Gender			
Male	215 (50.9)	52	0.67
Female	207 (49.1)	48	
Language ^b^			
English	280 (82.1)	84.1	0.32
Other	61 (17.9)	15.9	
School type ^c^			
Government	276 (65.9)	62.8	<0.001
Catholic	125 (29.8)	22.7	
Independent	18 (4.3)	14.5	
Metropolitan	331 (78.4) ^c^	71	<0.001
Rural	88 (20.9)	29	
SEIFA score	Mean: 1049.8; SD: 87.2	Mean: 1000.0; SD: 100.0	<0.01 ^d^
Age groups	Proportion of children in each age group in study sample		
	Female ^e^	Male ^e^	Total ^e^
6 year-olds	16	17	33
7 year-olds	19	20	39
8 year-olds	18	23	41
9 year-olds	19	20	39
10 year-olds	19	14	33
11 year-olds	29	29	58
12 year-olds	28	20	48
13 year-olds	19	16	35
14 year-olds	19	18	37
15 year-olds	8	8	16
16 year-olds	8	11	19
17 year-olds	9	7	16
18 year-olds	4	4	8

^a^ statistical differences between study group and state population (from Victorian summary statistics, where p ≥ 0.05 indicates that the groups have similar distributions for the variables (determined using chi-squared statistics); ^b^ language spoken at home - *n* = 81 missing data; ^c^ *n* = 3 missing data; ^d^ the distribution of SEIFA scores for the study participants and the State population data were compared using a One-Sample Wilcoxon Signed Rank Test; ^e^ indicates number in each age group for male and female children and the total number. F = female; M = male; SD = standard deviation; SEIFA = Socio-Economic Index for Area

**Fig. 1:**
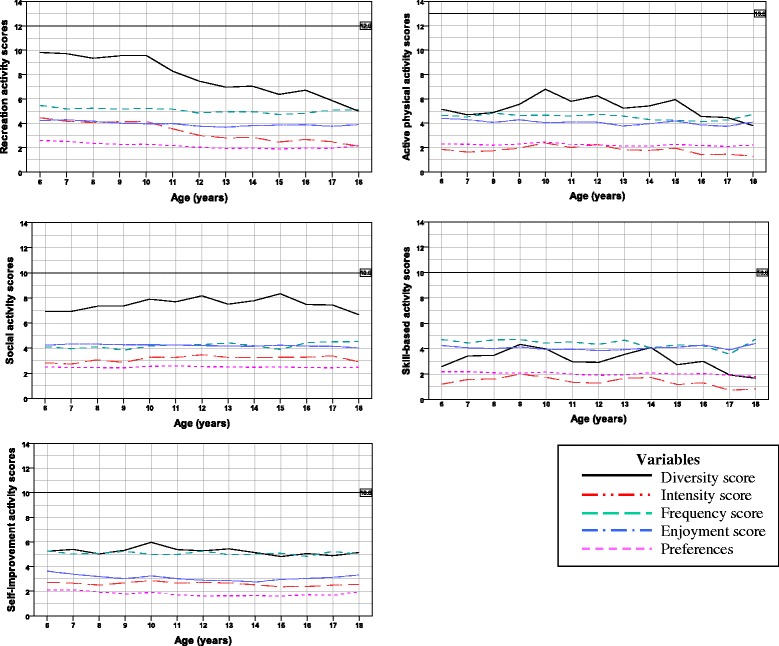
Mean activity scores according to activity type and age group. The reference line at the top of the graph shows the maximum diversity score possible for this activity domain. The range of possible scores for the rest of the items are as follows: intensity: 0.0–7.0; frequency: 0.0–7.0; enjoyment: 1.0–5.0; preferences: 1.0–3.0

Summary data for each participation domain, stratified by gender and age, are presented according to the five activity types in the Additional files 2, 3, 4, 5 and 6. The results of the curve estimation for the total dataset and when data were stratified by gender are presented in Table 2. Variation in the diversity and enjoyment scores for the five activity types was found across age, with relative stability in intensity, frequency and preference (Fig. 1). Visual inspection of Fig. 1 shows a pattern of changing diversity scores mirrored in the enjoyment scores, suggesting a relationship between enjoyment and number of activities undertaken in a particular activity type. Across each activity type, there was less variation in preference, intensity and frequency scores suggesting relative stability in these variables across the age range of 6 to 18 years (see Fig. 1).

**Table 2:** Curve estimation and regression coefficients for the overall diversity scores by activity type and gender

Activity type	Model type	Overall model			Comparison between genders					
					Males			Females		
		β (SE β)	*p* ^a^	R^2^ (*p* ^b^)	β (SE β)	*p* ^a^	R^2^ (*p* ^b^)	β (SE β)	*p* ^a^	R^2^ (*p* ^b^)
Recreational	Linear									
	Age	-0.42 (.03)	<.01	.26 (<.01)	-0.41 (.05)	<.01	.24 (<.01)	-0.41 (.05)	<.01	.29 (<.01)
	Constant	12.78 (.39)			12.34 (.57)			13.16 (.51)		
	Quadratic									
	Age	-0.16 (.23)	.49	.27 (<.01)	<0.01 (.33)	.99	.25 (<.01)	-0.26 (.31)	.40	.29 (<.01)
	Age^2^	-0.01 (.01)	.26		-0.02 (.01)	.20		-0.01 (.01)	.62	
	Constant	11.44 (1.24)			10.17 (1.79)			12.37 (1.67)		
Active-physical	Linear									
	Age	-0.03 (.04)	.43	<.01 (.43)	<-0.01 (.05)	.98	<.01 (.98)	-0.06 (.05)	.24	.01 (.24)
	Constant	5.69 (.42)			5.69 (.60)			5.72 (.58)		
	Quadratic									
	Age	1.25 (.24)	<.01	.07 (<.01)	0.97 (.34)	<.01	.04 (.01)	1.51 (.34)	<.01	.11 (<.01)
	Age^2^	-0.06 (.01)	<.01		-0.04 (.02)	<.01		-0.07 (.02)	<.01	
	Constant	-1.02 (1.30)			0.58 (1.84)			-2.44 (1.81)		
Social	Linear									
	Age	0.05 (.03)	.09	.01 (.09)	0.10 (.05)	.04	.02 (.04)	0.02 (.04)	.68	<.01 (.68)
	Constant	6.97 (.35)			6.05 (.53)			7.81 (.42)		
	Quadratic									
	Age	0.76 (.20)	<.01	.04 (<.01)	0.89 (.30)	<.01	.05 (<.01)	0.72 (.25)	<.01	.04 (.02)
	Age^2^	-0.03 (.01)	<.01		-0.04 (.01)	<.01		-0.03 (.01)	<.01	
	Constant	3.25 (1.09)			1.87 (1.63)			4.17 (1.36)		
Skill-Based	Linear									
	Age	-0.08 (.031)	<.01	.02 (<.01)	0.01 (.04)	.89	<.01 (.89)	-0.16 (.05)	.01	.05 (.01)
	Constant	3.93 (.36)			2.36 (.41)			5.38 (.54)		
	Quadratic									
	Age	0.59 (.21)	<.01	.04 (<.01)	0.53 (.23)	.02	.02 (.07)	0.77 (.32)	.02	.09 (<.01)
	Age^2^	-0.03 (.01)	<.01		-0.02 (.01)	.02		-0.04 (.01)	<.01	
	Constant	0.44 (1.13)			-0.40 (1.27)			0.55 (1.72)		
Self-Improvement	Linear									
	Age	-0.03 (.03)	.25	<.01 (.25)	0.03 (.04)	.46	<.01 (.46)	-0.08 (.04)	.02	.03 (.02)
	Constant	5.59 (.31)			4.41 (.43)			6.68 (.39)		
	Quadratic									
	Age	0.28 (.18)	.12	.01 (.11)	0.45 (.24)	.06	.02 (.16)	0.20 (.24)	.40	.03 (.04)
	Age^2^	-0.01 (.01)	.08		-0.02 (.01)	.08		-0.01 (.01)	.24	
	Constant	3.98 (.98)			2.17 (1.34)			5.23 (1.28)		

^a^ *p*-value for individual coefficients in the model. ^b^ *p*-value for the model itself

There was an apparent reduction in the number of recreational activities, and the enjoyment of those activities as children got older (see Fig. 1 and Additional files 2 and 5). Curve estimation suggested that a linear model best described the differences in recreational diversity over time (*p* < .01), with an overall decrease in diversity scores with age. Similar patterns were found when the data were stratified by gender (see Table 2).

As displayed in Table 2, diversity of active physical participation appeared to vary with age. Curve estimation suggested a quadratic model best described the pattern of participation for the grouped data (*p* < .01), including when the data were stratified by gender (males *p* = .01, females *p* < .01), with an initial increase in diversity during the primary school years followed by a decrease during the adolescent years. The patterns of participation differed between males and females, with female participants experiencing a greater decrease in the diversity of participation in active physical activities compared with males, during the years of 16 to 18 years.

Data on participation in social activities suggested an overall increase in diversity and enjoyment through the middle-teenage years, with consistent high levels of enjoyment (see Fig. 1 and Additional files 2 and 5). Accordingly, a quadratic approach best described the pattern of participation for the diversity of social activities (*p* < .01). When the data were stratified by gender, a quadratic approach remained the best model for females (*p* = .02) while a linear approach could explain the diversity scores for male participants (*p* = .04) with a small increase in the number of social activities with age (*β* = 0.10) (see Table 2).

The pattern of participation in skill-based and self-improvement activities across the age range of 6 to 18 years did not appear to demonstrate an obvious pattern of change according to Fig. 1, although peaks of activity were apparent in the 9 to 10, and 14 year old age groups. Curve estimation of skill-based diversity scores suggested a linear model would best describe the data (*p* < .01), with small decreases in the number of activities performed with age (*β* = -0.08). When the skill-based diversity data were split by gender, a linear model was also found to best explain the data for female participants (*p* < .01, *β* = -0.16), while neither a linear or a quadratic approach was found to fit the data for males (*p* = .89 and .07 respectively; see Table 2). Analysis of the diversity data for self-improvement activities was similar; a linear approach best described the data for female participants (*p* = .02, *β* = -0.08), while neither a linear or a quadratic approach could describe the patterns of participation for the grouped data (*p* = .25 and .12 respectively) or for male participants (*p* = .46 and .16 respectively; see Table 2).

Additional file 7 displays the data related to where participation occurred. These data demonstrated a large amount of between-participant variability but relative consistency across age groups, and little difference between males and females. Most recreational and self-improvement activities were performed at home, while a larger proportion of active physical, social and skill-based activities were performed away from home. The companionship data (Additional file 8) also demonstrated between-participant variability. Overall, these data suggest that higher proportions of adolescents participated in activities within each type alone, rather than with others, in comparison to the younger age groups.

## Discussion

### Representativeness of the sample

This study aimed to provide descriptive data from a representative sample of Australian children and adolescents living in Victoria to describe and enhance our understanding of their participation during outside school hours. The extent to which a representative sample was achieved was limited to gender and English language distribution. Although the absolute differences between population statistics and sample statistics were not large, the sample had a lower proportion of students from rural and independent schools, and a higher SEIFA score. The level of representativeness is likely to have been influenced by the very low consent rate (5.5 %) that resulted in some schools only contributing data from a small number of students and necessitated a higher number of schools to be approached. This raises questions about who the participants are; it is possible that the ‘high participators’ chose to take part. In addition, planned sample size estimates for the older age groups were not achieved and the adolescents were over-represented in terms of missing data. Recruiting older adolescents into the study was challenging: their interest in this study was not high and the format of the CAPE/PAC, which uses pictures, is less appropriate for this age group. The CAPE/PAC is valid for those aged 6 to 21 years; however a version without pictures would be valuable for the older group. The difference in sampling rates between older and younger participants also raises questions about consenting procedures in survey research involving children. Written consent in this study was obtained from both the parent and participant, however, it is possible that older adolescents felt more able to decline.

### Participation in activities outside school

Although there were difficulties achieving full representativeness, the study did include children and adolescents of both genders, across the targeted age range (6 to 18 years), from Government, Catholic and Independent schools, set in metropolitan and rural settings and from families with a range of socio-economic resources. The findings provide evidence of the participation patterns and preferences of the participants who can be considered as typically developing. Variations across age and gender in recreational, active-physical, social, skill-based and self-improvement activities completed outside mandated school were demonstrated. Although the data are cross-sectional and not longitudinal, they provide evidence of the extent to which participation patterns vary with age and where they remain stable. They also reveal potential relationships between participation dimensions. These data can be used by clinicians, educators, researchers and policy makers; the results provide evidence about patterns of participation in groups of children with typical development and therefore may be a helpful benchmark for comparison when planning future studies or programs for those with long term health conditions or disability. The findings may be particularly useful when tailoring programs to meet the needs of both males and females, as well as across different ages, and in consideration of the diversity of activities in which children and adolescents participate. The data may also be useful for those wishing to complete international comparisons to determine the effect of system, service or other environmental or cultural differences.

Although there are variations across age in each activity type and in each of the participation domains, there is evidence from these data that, on average, preferences for activity participation are relatively stable across the 6 to 18 year developmental period. This finding suggests that although participation in specific activities may wax and wane, preferences develop early and that children may acquire a taste for particular participation patterns very early in life. Research from the disability sector [34] indicates that preferences in particular activity types are strongly related to the extent of participation. Therefore, it is possible that interventions to influence patterns of participation may need to occur in young children, to establish healthy activity preferences.

There is clear and consistent evidence that participation diversity, intensity and preference for recreational activities is reduced in older children compared to younger [22, 34, 35]. This may reflect that some of the activities included in this domain, such as collecting things and colouring in, are likely to be replaced by other activities as children grow older. This change was also evident in our data. What is also apparent is that the frequency of participation in recreational activities (that is, how often you take part in the activities you do) showed little variation. Thus, it is important to measure both the number of activities and the frequency of attendance, to understand participation patterns. Taken together these data suggest a consistent amount of recreational participation throughout childhood and adolescence, although the actual activity and the number of activities may change.

In this study we derived a frequency score as well as an intensity score for each activity type. Intensity scores, calculated according to the CAPE manual, represent the average frequency of the total number of activities within a domain and are therefore measures of relative frequency [25]. Frequency scores were calculated within this study based on the average frequency of participation in the activities actually completed, thus providing a measure of average frequency of participation over the 4 month period. It is apparent that the intensity scores were relatively low in all activity types, and that the pattern of their variation across the age groups was in line with the variation in diversity scores, likely due to how they are calculated. This suggests changes in number of activities over time are well represented by the diversity score, and changes in frequency of participation are more clearly understood by calculating a frequency, rather than intensity score. The measure of frequency of participation within each activity domain showed little variation with age, suggesting that on average children and youth participated with consistent moderate to high frequency (score ranges between 3.55 and 5.47 out of 7).

Gender effects were apparent. Overall, female participants typically took part in more activities (higher diversity scores), more intensely, with higher enjoyment and had higher preferences for each activity type than males, with the exception of active physical activities. There were, however, also gender differences in the frequency of participation, with males taking part more often in self-improvement as well as active physical activities than females. These findings are consistent with past research that participation in sports is higher for males than females [26, 35], but differs from previous reports that females participate to a greater extent in self-improvement activities than males [23]. This difference highlights what can be learned from an assessment of frequency of participation as opposed to intensity and diversity: data from this study suggests that although males might undertake fewer self-improvement activities than females, the ones they do take part in, they do more often.

Gender differences were also apparent in the context in which participation took place. Female participants were less likely to participate alone than males, particularly in recreational activities, such as playing computer games, playing with toys and watching television. In relation to location, female participants were more likely than males to complete social activities at home, such as talking on the phone, listening to music, making food and entertaining others.

Curve estimation of the diversity scores also suggested potential gender differences in the age-related pattern of participation in skill-based and self-improvement activities: female participants demonstrated a linear trend with participation in fewer activities in the older age groups, but the pattern of participation for male participants could not easily be explained using a linear or quadratic approach. For each activity type, except self-improvement activities, it appeared there was a general decrease in diversity scores for females between the ages of 16 and 18 years. It is possible that with a larger sample of older children different patterns of participation for diversity scores may have been found based on age and gender.

### The measure chosen to assess participation

The original version of the CAPE/PAC is scored by calculating the means (SD) of six participation dimensions: diversity, intensity, with whom, where, enjoyment and preference, despite the original items being ordinal or categorical. In this study, we justify treating the data as continuous for four of those dimensions on the basis of past validation studies and in line with classical test theory approaches to measurement [36]. This approach, however, is not justifiable for the ‘where’ (location) and ‘with whom’ (companionship) scales as the items are categorical and not ordinal; therefore an alternate method was used to summarise these data in this study [24, 37]. Reporting proportions of children and adolescents who participate at home or away from home, and on their own or with others, is justified statistically, however does limit the level of detail that might be learned if further development of these scales was completed.

There is evidence to suggest that young children, in particular those younger than 8 years old, may have difficulty with recalling activities and accurately choosing a response when there are more than three categories [38]. Nevertheless, the importance of including self-report data, especially when considering preferences and enjoyment of children, has also been highlighted in the literature [38]. While the reliability and accuracy of responses provided by the young participants in this study was not assessed, it was interesting to note that the majority of the missing data were from participants in the older age groups. This might reflect that parental or teacher guidance was provided where needed to the younger children, and/or that the adolescents who completed the questionnaires alone tired of doing so and thus skipped items. It is possible that the younger participants in this study did not provide an accurate report of their participation, or that their responses were influenced strongly by parents, and thus reflect parent perception rather than child report; the extent to which this occurred is not known. Although caution when interpreting the results is warranted, the CAPE/PAC have been validated in this younger age group [15].

Measuring participation is difficult. Although the self-report nature of the CAPE/PAC questionnaires might be seen as a limitation, their strengths lies in that they were specifically designed to measure the construct of participation. The questionnaires provide rich data by describing multiple aspects of participation; and experiential aspects of participation (i.e. those related to involvement such as enjoyment and preference) can only be measured through self-report. Other methods, such as motion sensors, might be useful in supplementing data from the CAPE/PAC by measuring attendance or the amount of physical activity a person performs, but they cannot replace self-report data.

### Limitations

In this study participants from rural and independent schools, and those whose families had fewer resources, although sampled, were under-represented; thus there are limitations to the generalisability of the findings to those groups, and of the findings beyond Victoria. It could be argued that variations in geographical location (and potential differences in weather patterns) might impact on participation levels, and therefore, that results may only be generalisable to the southern parts of the state, where participants were located. However, it is also relevant to note that comparative studies between Australia, Canada and the US, involving samples of children with cerebral palsy, found very few geographical variations in CAPE/PAC scores [14], suggesting that season and/or weather is not likely to be a factor, because of the nature of the activity type variables. For example, children might change the sport undertaken between winter and summer, but they still do a sport, hence the CAPE score does not change.

## Conclusions

Data presented in this study provides evidence about the participation patterns outside school of children and adolescents aged 6 to 18 years. The findings suggest that participation patterns, as defined by the range, frequency, enjoyment of, and preference for activities completed outside school, are relatively stable across childhood. Future research investigating the determinants of participation in a population of children and adolescents without long term health conditions, will assist in understanding who are likely to be the high and low participators. This knowledge will further enhance our understanding of this complex construct and may inform policy makers and service providers when determining needs and supporting the participation outcomes of all children and youth.

## Abbreviations

ABS, Australian Bureau of Statistics; CAPE, Children’s Assessment of Participation and Environment; ICC, Intraclass correlation coefficient; ICF/ICF-CY, International Classification of Functioning Disability and Health/International Classification of Functioning Disability and Health-Children and Youth Version; PAC, Preferences for Activities of Children; SD, Standard deviation; SEIFA, Socio Economic Index for Areas.

## Additional files

Additional file 1: Demographic questionnaire. (PDF 36 kb) Additional file 2: Participation diversity in typically developing children according to activity type, age and gender. (DOCX 13 kb) Additional file 3: Frequency of participation in typically developing children according to activity type, age and gender. (DOCX 13 kb) Additional file 4: Participation intensity in typically developing children according to activity type, age and gender. (DOCX 13 kb) Additional file 5: Participation enjoyment in typically developing children according to activity type, age and gender. (DOCX 13 kb) Additional file 6: Participation preferences in typically developing children according to activity type, age and gender. (DOCX 13 kb) Additional file 7: Proportion (%) of activities performed at home according to activity type, age and gender. (DOCX 13 kb) Additional file 8: Proportion (%) of activities performed alone according to activity type, age and gender. (DOCX 14 kb)
