# Supplementary material for: A descriptive study of the participation of children and adolescents in activities outside school
Source: BMC Pediatr. 2016 Jul 8;16:84. doi: 10.1186/s12887-016-0623-9 (PMC4939009; doi:10.1186/s12887-016-0623-9)
Supplement: Additional file 1: — Demographic questionnaire. (PDF 36 kb) [file 12887_2016_623_MOESM1_ESM.pdf]

Demographic Questionnaire

The information provided by this questionnaire will assist in the collation and analysis of data from the CAPE and PAC. The following questions are to be completed by the child and/or the child's parents/guardians.

Name of person completing this questionnaire: ___________________________________
Relationship to the child (if applicable): ____

1. Participant's name: ________________________________
2. Age: ___________
3. Gender: M / F (please circle)
4. Weight (this information will be used to calculate your Body Mass Index):

   ___________________kg (please ensure measurements are as recent and accurate as possible)

   Height (this information will be used to calculate your Body Mass Index):

   ___________________ m (please ensure measurements are as recent and accurate as possible)
5. School: _________________________________
6. Year level: _________________________________
7. Postcode of school: ______________________________________
8. Country of birth: ___________________________________(participant)

   _____________________________(parent/guardian)
9. Other languages spoken (other than English): __________________________
10. Have there been any major changes or events for the child and/or family in the past four months that may have affected the child's level of participation? (eg. a significant illness, a long vacation or relocation) ____
    ____
    ____

11. Does the child require an integration aid to participate in school activities? If so, in which activities?

    ____
    ____
