# Supplementary material for: A descriptive study of the participation of children and adolescents in activities outside school
Source: BMC Pediatr. 2016 Jul 8;16:84. doi: 10.1186/s12887-016-0623-9 (PMC4939009; doi:10.1186/s12887-016-0623-9)
Supplement: Additional file 2: — Participation diversity in typically developing children according to activity type, age and gender. (DOCX 13 kb) [file 12887_2016_623_MOESM2_ESM.docx]

Additional file 2: Participation diversity in typically developing children according to activity type, age and gender

|  | **Recreational** | **Active Physical** | **Social** | **Skill-Based** | **Self-Improvement** | **Formal** | **Informal** |
| --- | --- | --- | --- | --- | --- | --- | --- |
| Overall | 8.22 (2.57) | 5.37 (2.39) | 7.54 (1.98) | 3.03 (2.04) | 5.25 (1.75) | 4.33 (2.43) | 25.09 (5.92) |
| Male | 7.80 (2.60) | 5.68 (2.36) | 7.11 (2.11) | 2.41 (1.62) | 4.71 (1.70) | 3.91 (2.07) | 23.80 (5.91) |
| Female | 8.66 (2.47) | 5.05 (2.38) | 7.98 (1.72) | 3.67 (2.24) | 5.82 (1.62) | 4.76 (2.69) | 26.42 (5.64) |
| 6yo | 9.82 (1.74) | 5.15 (2.12) | 6.91 (2.10) | 2.42 (1.46) | 5.24 (1.52) | 3.42 (1.87) | 26.12 (4.99) |
| 7yo | 9.72 (2.01) | 4.56 (2.23) | 6.92 (2.50) | 3.33 (2.17) | 5.26 (2.01) | 4.10 (2.27) | 25.69 (6.64) |
| 8yo | 9.34 (2.04) | 4.88 (1.91) | 7.34 (1.92) | 3.39 (2.11) | 5.02 (1.37) | 4.66 (2.25) | 25.31 (5.41) |
| 9yo | 9.54 (1.97) | 5.41 (2.26) | 7.36 (2.31) | 4.00 (2.27) | 5.33 (1.96) | 5.36 (2.53) | 26.28 (6.57) |
| 10yo | 9.58 (2.08) | 6.79 (2.37) | 8.00 (2.00) | 3.73 (2.02) | 5.97 (1.99) | 5.30 (2.28) | 28.76 (6.08) |
| 11yo | 8.28 (1.94) | 5.79 (2.18) | 7.69 (1.72) | 2.55 (2.02) | 5.38 (1.77) | 4.07 (2.32) | 25.62 (4.73) |
| 12yo | 7.46 (2.74) | 6.29 (2.27) | 8.17 (1.62) | 2.85 (1.57) | 5.31 (1.46) | 4.48 (2.10) | 25.60 (5.22) |
| 13yo | 6.91 (2.43) | 5.23 (2.53) | 7.57 (1.96) | 3.09 (2.19) | 5.23 (1.73) | 4.57 (2.75) | 23.46 (6.21) |
| 14yo | 7.05 (2.31) | 5.27 (2.41) | 7.78 (1.67) | 3.65 (2.26) | 5.14 (1.97) | 4.78 (2.75) | 24.11 (5.37) |
| 15yo | 6.38 (2.78) | 5.94 (2.57) | 8.13 (1.54) | 2.56 (1.90) | 4.81 (1.87) | 3.81 (2.59) | 24.00 (6.77) |
| 16yo | 6.53 (2.46) | 4.53 (2.34) | 7.47 (1.87) | 2.37 (1.64) | 5.05 (1.78) | 3.79 (2.30) | 22.16 (5.43) |
| 17yo | 5.75 (1.44) | 4.19 (2.43) | 7.44 (1.86) | 1.69 (1.45) | 4.88 (1.31) | 2.63 (2.03) | 21.31 (4.14) |
| 18yo | 4.63 (3.02) | 2.50 (2.98) | 6.00 (2.14) | 1.25 (1.28) | 4.63 (2.00) | 1.88 (2.23) | 17.13 (6.33) |

Note: Items are scored: have done: yes = 1; have not done: no = 0). Maximum possible scores are Recreational = 12; Active-Physical = 13; Social = 10; Skill-Based = 10; Self-Improvement = 10; Formal = 15; Informal = 40. All data are presented as mean (SD) for each age group/activity type.
