# Supplementary material for: A descriptive study of the participation of children and adolescents in activities outside school
Source: BMC Pediatr. 2016 Jul 8;16:84. doi: 10.1186/s12887-016-0623-9 (PMC4939009; doi:10.1186/s12887-016-0623-9)
Supplement: Additional file 3: — Frequency of participation in typically developing children according to activity type, age and gender. (DOCX 13 kb) [file 12887_2016_623_MOESM3_ESM.docx]

Additional file 3: Frequency of participation in typically developing children according to activity type, age and gender

|  | **Recreational** | **Active Physical** | **Social** | **Skill-Based** | **Self-Improvement** | **Formal** | **Informal** |
| --- | --- | --- | --- | --- | --- | --- | --- |
| Overall | 5.10 (0.81) | 4.57 (0.97) | 4.17 (0.86) | 4.46 (1.23) | 5.08 (0.86) | 4.56 (0.99) | 4.71 (0.59) |
| Male | 5.07 (0.82) | 4.69 (0.90) | 4.04 (0.83) | 4.34 (1.36) | 5.11 (0.91) | 4.64 (0.99) | 4.66 (0.58) |
| Female | 5.12 (0.81) | 4.44 (1.02) | 4.31 (0.86) | 4.57 (1.08) | 5.04 (0.80) | 4.47 (0.98) | 4.75 (0.60) |
| 6yo | 5.47 (0.56) | 4.65 (1.11) | 4.11 (1.12) | 4.71 (1.02) | 5.27 (0.76) | 4.84 (0.90) | 4.94 (0.60) |
| 7yo | 5.18 (0.85) | 4.52 (0.95) | 3.95 (0.79) | 4.45 (1.06) | 5.02 (0.95) | 4.58 (1.05) | 4.72 (0.68) |
| 8yo | 5.24 (0.77) | 4.83 (1.00) | 4.10 (0.98) | 4.69 (1.07) | 5.07 (0.90) | 4.62 (0.78) | 4.82 (0.59) |
| 9yo | 5.18 (0.76) | 4.64 (0.94) | 3.84 (1.00) | 4.72 (1.00) | 5.24 (1.00) | 4.66 (0.89) | 4.69 (0.61) |
| 10yo | 5.22 (0.72) | 4.66 (0.94) | 4.16 (0.73) | 4.44 (1.23) | 4.98 (0.85) | 4.72 (0.93) | 4.73 (0.57) |
| 11yo | 5.16 (0.76) | 4.58 (1.03) | 4.25 (0.74) | 4.52 (1.27) | 4.97 (0.86) | 4.50 (0.92) | 4.73 (0.56) |
| 12yo | 4.84 (0.87) | 4.73 (0.75) | 4.28 (0.80) | 4.34 (1.31) | 5.26 (0.86) | 4.70 (0.86) | 4.67 (0.59) |
| 13yo | 4.95 (0.86) | 4.59 (0.98) | 4.41 (0.68) | 4.64 (1.02) | 4.98 (0.73) | 4.80 (0.93) | 4.67 (0.50) |
| 14yo | 4.96 (0.88) | 4.30 (0.96) | 4.19 (0.64) | 4.06 (1.57) | 5.01 (0.69) | 4.13 (1.09) | 4.56 (0.49) |
| 15yo | 4.72 (0.79) | 4.25 (0.86) | 3.98 (0.98) | 4.31 (1.12) | 5.09 (0.90) | 4.77 (0.96) | 4.38 (0.60) |
| 16yo | 4.84 (1.10) | 4.13 (0.99) | 4.43 (0.90) | 4.23 (1.48) | 4.83 (1.05) | 4.14 (1.02) | 4.52 (0.71) |
| 17yo | 5.08 (0.70) | 4.26 (1.03) | 4.50 (0.84) | 3.56 (1.71) | 5.24 (0.77) | 3.55 (1.57) | 4.82 (0.48) |
| 18yo | 5.20 (0.53) | 4.72 (0.81) | 4.40 (0.95) | 4.75 (1.08) | 4.98 (0.67) | 4.93 (0.72) | 4.74 (0.56) |

Note: Items are scored: 1 = 1 time in past 4 months; 2 = 2 times in past 4 months; 3 = 1 time a month; 4 = 2-3 times a month; 5 = 1 time a week; 6 = 2-3 times a week; 7 = 1 time a day or more. Frequency is calculated as the average frequency divided by the total number of activities undertaken by the participant in the four month period, with a maximum possible score for all activity types of 7. All data are presented as mean (SD) for each age group/activity type.
