# Supplementary material for: A descriptive study of the participation of children and adolescents in activities outside school
Source: BMC Pediatr. 2016 Jul 8;16:84. doi: 10.1186/s12887-016-0623-9 (PMC4939009; doi:10.1186/s12887-016-0623-9)
Supplement: Additional file 4: — Participation intensity in typically developing children according to activity type, age and gender. (DOCX 13 kb) [file 12887_2016_623_MOESM4_ESM.docx]

Additional file 4: Participation intensity in typically developing children according to activity type, age and gender

|  | **Recreational** | **Active Physical** | **Social** | **Skill-Based** | **Self-Improvement** | **Formal** | **Informal** |
| --- | --- | --- | --- | --- | --- | --- | --- |
| Overall | 3.51 (1.22) | 1.89 (0.87) | 3.15 (1.00) | 1.48 (0.96) | 2.63 (0.81) | 1.32 (0.76) | 2.95 (0.75) |
| Male | 3.30 (1.19) | 2.02 (0.91) | 2.89 (1.03) | 1.20 (0.74) | 2.36 (0.74) | 1.21 (0.66) | 2.76 (0.74) |
| Female | 3.72 (1.21) | 1.75 (0.81) | 3.43 (0.87) | 1.76 (1.07) | 2.90 (0.78) | 1.43 (0.84) | 3.14 (0.71) |
| 6yo | 4.46 (0.89) | 1.85 (1.02) | 2.83 (0.97) | 1.20 (0.67) | 2.73 (0.83) | 1.08 (0.62) | 3.21 (0.65) |
| 7yo | 4.18 (1.03) | 1.63 (0.82) | 2.74 (1.14) | 1.57 (1.13) | 2.65 (0.92) | 1.25 (0.80) | 3.01 (0.83) |
| 8yo | 4.07 (1.03) | 1.74 (0.63) | 3.08 (1.12) | 1.60 (1.02) | 2.50 (0.71) | 1.40 (0.65) | 3.05 (0.73) |
| 9yo | 4.13 (1.07) | 1.96 (0.85) | 2.86 (1.24) | 1.99 (0.98) | 2.68 (0.91) | 1.65 (0.78) | 3.09 (0.89) |
| 10yo | 4.15 (1.01) | 2.38 (0.86) | 3.32 (1.01) | 1.74 (0.86) | 2.88 (0.84) | 1.61 (0.66) | 3.37 (0.73) |
| 11yo | 3.55 (0.97) | 2.01 (0.83) | 3.26 (0.89) | 1.33 (0.99) | 2.66 (0.88) | 1.24 (0.73) | 3.03 (0.67) |
| 12yo | 3.02 (1.30) | 2.26 (0.86) | 3.47 (0.85) | 1.28 (0.75) | 2.70 (0.63) | 1.37 (0.66) | 2.98 (0.69) |
| 13yo | 2.79 (0.91) | 1.81 (0.84) | 3.28 (0.77) | 1.68 (0.94) | 2.65 (0.73) | 1.42 (0.84) | 2.71 (0.65) |
| 14yo | 2.86 (0.96) | 1.76 (0.83) | 3.25 (0.81) | 1.73 (1.09) | 2.51 (0.90) | 1.34 (0.92) | 2.74 (0.63) |
| 15yo | 2.47 (1.07) | 1.95 (0.94) | 3.23 (1.01) | 1.18 (0.93) | 2.36 (0.75) | 1.20 (0.89) | 2.60 (0.75) |
| 16yo | 2.68 (0.99) | 1.42 (0.85) | 3.28 (0.88) | 1.31 (0.80) | 2.38 (0.77) | 1.00 (0.70) | 2.50 (0.72) |
| 17yo | 2.48 (0.67) | 1.46 (0.80) | 3.38 (1.09) | 0.73 (0.60) | 2.49 (0.52) | 0.68 (0.58) | 2.56 (0.51) |
| 18yo | 2.15 (1.26) | 1.29 (0.94) | 2.81 (0.55) | 0.82 (0.70) | 2.57 (0.89) | 0.95 (0.67) | 2.21 (0.64) |

Note: Items are scored: 1 = 1 time in past 4 months; 2 = 2 times in past 4 months; 3 = 1 time a month; 4 = 2-3 times a month; 5 = 1 time a week; 6 = 2-3 times a week; 7 = 1 time a day or more. Intensity is calculated as the average frequency divided by the total possible number of activities in the scale with a maximum possible score for all activity types of 7. All data are presented as mean (SD) for each age group/activity type.
