# Supplementary material for: A descriptive study of the participation of children and adolescents in activities outside school
Source: BMC Pediatr. 2016 Jul 8;16:84. doi: 10.1186/s12887-016-0623-9 (PMC4939009; doi:10.1186/s12887-016-0623-9)
Supplement: Additional file 5: — Participation enjoyment in typically developing children according to activity type, age and gender. (DOCX 13 kb) [file 12887_2016_623_MOESM5_ESM.docx]

Additional file 5: Participation enjoyment in typically developing children according to activity type, age and gender

|  | **Recreational** | **Active Physical** | **Social** | **Skill-Based** | **Self-Improvement** | **Formal** | **Informal** |
| --- | --- | --- | --- | --- | --- | --- | --- |
| Overall | 3.97 (0.54) | 4.08 (0.63) | 4.23 (0.49) | 4.02 (0.83) | 3.09 (0.79) | 4.08 (0.67) | 3.89 (0.45) |
| Male | 3.94 (0.56) | 4.08 (0.64) | 4.14 (0.52) | 3.78 (0.91) | 2.85 (0.83) | 4.00 (0.70) | 3.81 (0.47) |
| Female | 4.00 (0.53) | 4.08 (0.62) | 4.33 (0.45) | 4.26 (0.67) | 3.33 (0.66) | 4.16 (0.64) | 3.98 (0.40) |
| 6yo | 4.24 (0.44) | 4.39 (0.63) | 4.24 (0.52) | 4.25 (0.90) | 3.63 (0.89) | 4.31 (0.79) | 4.15 (0.41) |
| 7yo | 4.29 (0.52) | 4.30 (0.60) | 4.34 (0.58) | 4.07 (1.00) | 3.37 (0.86) | 4.08 (0.77) | 4.14 (0.48) |
| 8yo | 4.18 (0.47) | 4.07 (0.73) | 4.32 (0.578) | 4.00 (0.91) | 3.18 (0.73) | 3.96 (0.77) | 4.02 (0.46) |
| 9yo | 4.00 (0.44) | 4.28 (0.53) | 4.27 (0.43) | 4.13 (0.71) | 3.01 (0.84) | 4.17 (0.60) | 3.94 (0.42) |
| 10yo | 3.97 (0.55) | 4.04 (0.53) | 4.29 (0.39) | 3.93 (0.87) | 3.25 (0.73) | 4.02 (0.72) | 3.92 (0.40) |
| 11yo | 3.98 (0.53) | 4.10 (0.59) | 4.25 (0.54) | 3.96 (0.78) | 3.02 (0.78) | 4.10 (0.64) | 3.86 (0.46) |
| 12yo | 3.76 (0.50) | 4.08 (0.56) | 4.21 (0.41) | 3.85 (0.91) | 2.89 (0.69) | 4.10 (0.48) | 3.76 (0.40) |
| 13yo | 3.71 (0.57) | 3.78 (0.75) | 4.17 (0.44) | 3.92 (0.84) | 2.86 (0.58) | 3.99 (0.68) | 3.67 (0.38) |
| 14yo | 3.82 (0.50) | 3.95 (0.53) | 4.15 (0.37) | 4.05 (0.59) | 2.74 (0.62) | 4.03 (0.61) | 3.74 (0.34) |
| 15yo | 3.86 (0.60) | 4.19 (0.46) | 4.23 (0.62) | 4.11 (0.79) | 2.95 (0.77) | 4.25 (0.71) | 3.85 (0.46) |
| 16yo | 3.89 (0.51) | 3.85 (0.58) | 4.16 (0.52) | 4.19 (0.67) | 3.04 (0.72) | 4.03 (0.63) | 3.81 (0.37) |
| 17yo | 3.77 (0.58) | 3.74 (0.58) | 4.15 (0.51) | 3.90 (0.80) | 3.12 (0.94) | 3.71 (0.63) | 3.79 (0.48) |
| 18yo | 3.93 (0.76) | 3.75 (1.09) | 3.92 (0.65) | 4.42 (0.92) | 3.28 (0.77) | 4.30 (0.92) | 3.66 (0.62) |

Note: Item are scored 1 = not at all; 2 = somewhat sort of; 3 = pretty much; 4 = very much; 5 = love it. Maximum possible enjoyment score for all activity types is 5. All data are presented as mean (SD) for each age group/activity type.
