# Supplementary material for: A descriptive study of the participation of children and adolescents in activities outside school
Source: BMC Pediatr. 2016 Jul 8;16:84. doi: 10.1186/s12887-016-0623-9 (PMC4939009; doi:10.1186/s12887-016-0623-9)
Supplement: Additional file 6: — Participation preferences in typically developing children according to activity type, age and gender. (DOCX 13 kb) [file 12887_2016_623_MOESM6_ESM.docx]

Additional file 6: Participation preferences in typically developing children according to activity type, age and gender

|  | **Recreational** | **Active Physical** | **Social** | **Skill-Based** | **Self-Improvement** | **Formal** | **Informal** |
| --- | --- | --- | --- | --- | --- | --- | --- |
| Overall | 2.19 (0.40) | 2.23 (0.37) | 2.50 (0.34) | 2.00 (0.53) | 1.79 (0.44) | 1.96 (0.42) | 2.22 (0.30) |
| Male | 2.11 (0.39) | 2.27 (0.36) | 2.38 (0.37) | 1.73 (0.47) | 1.62 (0.41) | 1.81 (0.40) | 2.13 (0.30) |
| Female | 2.28 (0.40) | 2.18 (0.37) | 2.62 (0.25) | 2.27 (0.43) | 1.96 (0.40) | 2.12 (0.38) | 2.31 (0.28) |
| 6yo | 2.60 (0.38) | 2.29 (0.44) | 2.50 (0.41) | 2.14 (0.60) | 2.08 (0.46) | 2.10 (0.47) | 2.41 (0.37) |
| 7yo | 2.52 (0.39) | 2.27 (0.39) | 2.45 (0.39) | 2.17 (0.56) | 2.08 (0.48) | 2.12 (0.46) | 2.37 (0.33) |
| 8yo | 2.36 (0.33) | 2.20 (0.37) | 2.45 (0.31) | 2.09 (0.54) | 1.92 (0.46) | 2.03 (0.43) | 2.28 (0.31) |
| 9yo | 2.27 (0.37) | 2.28 (0.32) | 2.44 (0.41) | 2.06 (0.51) | 1.78 (0.38) | 2.06 (0.36) | 2.22 (0.30) |
| 10yo | 2.28 (0.37) | 2.46 (0.31) | 2.57 (0.27) | 2.12 (0.55) | 1.89 (0.45) | 2.12 (0.40) | 2.34 (0.30) |
| 11yo | 2.18 (0.29) | 2.26 (0.34) | 2.58 (0.31) | 1.92 (0.56) | 1.71 (0.42) | 1.89 (0.46) | 2.23 (0.26) |
| 12yo | 2.05 (0.30) | 2.21 (0.33) | 2.53 (0.30) | 1.89 (0.46) | 1.61 (0.33) | 1.86 (0.34) | 2.14 (0.23) |
| 13yo | 1.95 (0.37) | 2.14 (0.35) | 2.49 (0.33) | 1.88 (0.48) | 1.61 (0.35) | 1.83 (0.39) | 2.09 (0.23) |
| 14yo | 1.98 (0.32) | 2.11 (0.32) | 2.47 (0.34) | 2.01 (0.46) | 1.64 (0.38) | 1.90 (0.36) | 2.10 (0.24) |
| 15yo | 1.90 (0.37) | 2.26 (0.29) | 2.51 (0.37) | 1.94 (0.42) | 1.61 (0.32) | 1.92 (0.32) | 2.10 (0.26) |
| 16yo | 1.95 (0.40) | 2.15 (0.38) | 2.45 (0.33) | 1.85 (0.50) | 1.71 (0.35) | 1.86 (0.39) | 2.09 (0.31) |
| 17yo | 1.91 (0.37) | 2.06 (0.37) | 2.44 (0.36) | 1.82 (0.46) | 1.68 (0.44) | 1.82 (0.37) | 2.05 (0.28) |
| 18yo | 2.15 (0.29) | 1.90 (0.54) | 2.49 (0.18) | 1.69 (0.61) | 1.86 (0.63) | 1.68 (0.55) | 2.19 (0.30) |

Note: Items are scored: 1 = would not like to do at all; 2 = would sort of like to do; 3 = would really like to do. Maximum possible preference score for all activity types is 3. All data are presented as mean (SD) for each age group/activity type.
