# Supplementary material for: A descriptive study of the participation of children and adolescents in activities outside school
Source: BMC Pediatr. 2016 Jul 8;16:84. doi: 10.1186/s12887-016-0623-9 (PMC4939009; doi:10.1186/s12887-016-0623-9)
Supplement: Additional file 7: — Proportion (%) of activities performed at home according to activity type, age and gender. (DOCX 13 kb) [file 12887_2016_623_MOESM7_ESM.docx]

Additional file 7: Proportion (%) of activities performed at home according to activity type, age and gender

|  | **Recreational** | **Active Physical** | **Social** | **Skill-Based** | **Self-Improvement** | **Informal** | **Formal** |
| --- | --- | --- | --- | --- | --- | --- | --- |
| Overall | 73.42 (16.11) | 23.15 (21.86) | 37.06 (14.68) | 23.33 (29.11) | 64.69 (18.86) | 53.88 (10.85) | 11.18 (17.06) |
| Male | 73.80 (16.66) | 22.41 (21.71) | 34.71 (16.12) | 23.04 (29.82) | 67.52 (19.64) | 52.99 (11.07) | 11.88 (17.54) |
| Female | 73.02 (15.54) | 23.95 (22.04) | 39.51 (12.60) | 23.60 (28.47) | 61.79 (17.59) | 54.80 (10.56) | 10.44 (16.56) |
| 6yo | 74.95 (14.43) | 32.16 (25.18) | 34.59 (16.04) | 28.98 (29.55) | 67.47 (19.57) | 58.47 (12.14) | 7.79 (12.62) |
| 7yo | 75.13 (14.34) | 25.24 (23.71) | 38.50 (17.32) | 20.21 (24.70) | 61.54 (14.95) | 57.95 (9.04) | 10.10 (20.12) |
| 8yo | 74.18 (11.78) | 30.59 (23.78) | 38.23 (15.71) | 26.74 (31.75) | 62.80 (22.02) | 56.46 (9.94) | 15.17 (19.19) |
| 9yo | 69.19 (18.43) | 19.59 (18.18) | 28.96 (16.74) | 26.51 (26.98) | 65.37 (23.64) | 51.38 (14.56) | 14.09 (17.41) |
| 10yo | 70.83 (16.12) | 25.37 (22.46) | 33.34 (17.50) | 20.31 (23.31) | 63.97 (20.61) | 50.94 (12.91) | 13.39 (17.18) |
| 11yo | 71.54 (17.40) | 20.65 (19.25) | 39.82 (12.90) | 23.76 (34.58) | 64.42 (18.69) | 53.51 (10.20) | 9.45 (17.82) |
| 12yo | 72.21 (18.08) | 20.14 (16.85) | 38.48 (13.52) | 22.89 (31.35) | 65.55 (15.93) | 52.21 (10.53) | 9.11 (13.76) |
| 13yo | 75.45 (17.00) | 19.88 (20.82) | 35.36 (12.06) | 14.67 (20.12) | 69.70 (17.25) | 52.99 (8.31) | 7.52 (14.41) |
| 14yo | 77.77 (13.91) | 19.70 (19.96) | 37.59 (10.38) | 20.83 (21.87) | 67.78 (16.56) | 53.78 (7.49) | 11.08 (13.77) |
| 15yo | 72.01 (17.62) | 20.57 (19.45) | 36.55 (8.49) | 20.22 (30.98) | 63.68 (11.62) | 48.85 (8.82) | 12.93 (18.76) |
| 16yo | 71.38 (15.59) | 25.32 (27.92) | 42.18 (12.08) | 24.27 (30.34) | 60.11 (18.81) | 53.24 (8.56) | 12.31 (18.05) |
| 17yo | 75.63 (17.70) | 13.95 (17.20) | 42.94 (9.97) | 30.77 (39.00) | 61.28 (19.15) | 52.70 (11.55) | 11.55 (17.28) |
| 18yo | 81.22 (16.46) | 31.02 (44.67) | 42.72 (21.97) | 33.33 (51.64) | 58.65 (28.87) | 56.98 (14.94) | 26.67 (36.51) |

Note: Items were dichotomised to 1 = at home; 2 = away from home. All data are presented as mean (SD) for the percentage of activities performed at home for each age group/activity type.
