# Supplementary material for: A descriptive study of the participation of children and adolescents in activities outside school
Source: BMC Pediatr. 2016 Jul 8;16:84. doi: 10.1186/s12887-016-0623-9 (PMC4939009; doi:10.1186/s12887-016-0623-9)
Supplement: Additional file 8: — Proportion (%) of activities performed alone according to activity type, age and gender. (DOCX 14 kb) [file 12887_2016_623_MOESM8_ESM.docx]

Additional file 8: Proportion (%) of activities performed alone according to activity type, age and gender

|  | **Recreational** | **Active Physical** | **Social** | **Skill-Based** | **Self-Improvement** | **Formal** | **Informal** |
| --- | --- | --- | --- | --- | --- | --- | --- |
| Overall | 39.76 (22.29) | 18.10 (21.17) | 16.69 (13.51) | 17.68 (27.80) | 52.47 (25.07) | 12.73 (20.87) | 32.52 (13.75) |
| Male | 41.79 (22.36) | 19.55 (22.20) | 16.10 (13.58) | 19.94 (31.54) | 53.43 (27.43) | 13.49 (22.62) | 33.16 (13.90) |
| Female | 37.66 (22.07) | 16.56 (19.96) | 17.31 (13.44) | 15.47 (23.45) | 51.47 (22.42) | 11.93 (18.91) | 31.86 (13.60) |
| 6yo | 34.71 (20.74) | 9.15 (20.12) | 9.37 (10.92) | 14.46 (28.49) | 20.61 (15.75) | 10.25 (25.41) | 22.17 (10.91) |
| 7yo | 30.49 (19.21) | 10.84 (19.77) | 9.75 (13.87) | 18.95 (30.38) | 31.82 (24.09) | 17.99 (27.83) | 23.32 (11.98) |
| 8yo | 37.07 (19.74) | 11.68 (16.28) | 16.91 (17.08) | 22.75 (30.94) | 53.47 (22.79) | 16.32 (22.26) | 31.46 (15.32) |
| 9yo | 36.49 (16.38) | 10.09 (15.64) | 13.89 (13.53) | 14.68 (21.99) | 55.90 (26.72) | 11.01 (19.13) | 29.90 (11.64) |
| 10yo | 37.35 (18.84) | 19.74 (23.66) | 16.12 (14.45) | 14.67 (24.00) | 56.01 (26.90) | 13.67 (21.47) | 31.76 (14.64) |
| 11yo | 36.64 (21.64) | 19.00 (20.60) | 18.83 (14.35) | 14.87 (28.75) | 54.61 (20.00) | 8.09 (15.50) | 32.92 (13.63) |
| 12yo | 38.83 (21.38) | 22.09 (20.64) | 18.51 (12.42) | 16.44 (26.72) | 59.04 (20.56) | 10.32 (19.24) | 34.77 (12.26) |
| 13yo | 47.96 (23.52) | 18.32 (17.76) | 19.01 (12.40) | 19.11 (30.96) | 61.74 (18.94) | 10.09 (17.51) | 38.49 (13.15) |
| 14yo | 47.65 (25.27) | 23.42 (20.42) | 18.62 (10.15) | 19.36 (19.23) | 62.06 (21.09) | 11.53 (13.39) | 37.49 (12.32) |
| 15yo | 44.16 (20.02) | 29.23 (14.07) | 15.29 (9.41) | 20.83 (29.25) | 60.64 (22.61) | 20.94 (24.41) | 34.24 (7.80) |
| 16yo | 41.34 (25.39) | 21.54 (24.49) | 23.48 (9.92) | 16.25 (29.18) | 55.20 (19.96) | 9.49 (14.02) | 35.85 (9.24) |
| 17yo | 47.71 (26.54) | 36.64 (25.18) | 21.26 (8.40) | 23.81 (37.39) | 64.02 (22.24) | 22.72 (29.34) | 40.48 (14.16) |
| 18yo | 72.89 (28.21) | 38.43 (40.92) | 27.73 (13.85) | 25.00 (41.83) | 60.73 (31.56) | 48.75 (14.89) | 25.00 (27.64) |

Note: Items were dichotomised to 1 = alone; 2 = with others. All data are presented as mean (SD) for each for the percentage of activities performed alone for age group/activity type.
